# Estrogen Receptor Alpha Prevents Bladder Cancer Development via INPP4B inhibited Akt Pathway *in vitro* and *in vivo*

**DOI:** 10.18632/oncotarget.1421

**Published:** 2014-08-13

**Authors:** Iawen Hsu, Chiuan-Ren Yeh, Spencer Slavin, Hiroshi Miyamoto, George J. Netto, Mesut Muyan, Xue-Ru Wu, Edward M. Messing, Elizabeth A. Guancial, Shuyuan Yeh

**Affiliations:** ^1^ Departments of Urology and Pathology, University of Rochester Medical Center, Rochester, NY 14642; ^2^ Departments of Pathology, Urology, and Oncology, The Johns Hopkins Hospital, Baltimore, MD 21231; ^3^ Department of Biochemistry & Biophysics, University of Rochester Medical Center, Rochester, NY 14642; ^4^ Department of Urology, New York University, School of Medicine, NY 10016; ^5^ Departments of Hematology and Oncology, University of Rochester Medical Center, Rochester, NY 14642

## Abstract

Clinical reports show males have a higher bladder cancer (BCa) incidence than females. The sexual difference of BCa occurrence suggests that estrogen and its receptors may affect BCa development. Estrogen receptor alpha (ERα) is the classic receptor to convey estrogen signaling, however, the function of ERα in BCa development remains largely unknown. To understand the *in vivo* role of ERα in BCa development, we generated total and urothelial specific ERα knockout mice (ERαKO) and used the pre-carcinogen BBN to induce BCa. Earlier reports showed that ERα promotes breast and ovarian cancers in females. Surprisingly and of clinical importance, our results showed that ERα inhibits BCa development and loss of the ERα gene results in an earlier onset and higher incidence of BBN-induced *in vivo* mouse BCa. Supportively, carcinogen induced malignant transformation ability was reduced in ERα expressing urothelial cells as compared to ERα negative cells. Mechanism studies suggest that ERα could control the expression of INPP4B to reduce AKT activity and consequently reduce BCa cell growth. In addition, IHC staining of clinical sample analyses show that INPP4B expression, in correlation with reduced ERα, is significantly reduced in human BCa specimens. Together, this is the first report using the *in vivo* cre-loxP gene knockout mouse model to characterize ERα roles in BCa development. Our studies provide multiple *in vitro* cell studies and *in vivo* animal model data as well as human BCa tissue analyses to prove ERα plays a protective role in BCa initiation and growth at least partly via modulating the INPP4B/Akt pathway.

## INTRODUCTION

Urothelial carcinoma of the bladder is the fourth most common cancer in men and eleventh most common cancer in women in the United States [1]. It has been reported that BCa has the highest lifetime treatment cost per patient among all types of solid cancers [2], and most therapies for BCa patients will eventually fail. Thus, it is critical to understand the underlying mechanisms and find an approach to manage BCa development. Aging, environmental chemical exposure, and infectious parasites were found to be associated with higher BCa risks. Clinically, BCa incidence for males to females is 3.5 to 1 [3], suggesting sex hormone signals may play a role in the occurrence of BCa. Supportively, evidence suggests androgens/androgen receptor (AR) promote BCa development [4]. However, it remains unclear whether estrogen and estrogen receptors (ERs) play roles in the BCa occurrence.

There are two major types of estrogen receptors, ERα and ERβ, mediating estrogen effects in various tissues [5-9]. In addition, it has been known that estrogen and anti-estrogens will both activate a membrane protein, GPR30 [10, 11]. The roles of each ER in BCa development are understudied areas. In this report, we focused on investigating ERα roles in BCa development. ERα is a well-known transcriptional factor and belongs to the nuclear receptor superfamily. 17β-estradiol (E2), the natural ligand of ERα, can bind to and regulate ERα downstream gene expression. Multiple mechanisms have been proposed by which ERα can activate downstream gene expressions to exert its function [9]. In addition, growth factor signaling pathways such as EGF [12] or IGF-2 [13], can activate ERα in the absence of E2 in vascular or cancer cells.

The different levels of ERα expression in normal bladder tissues compared to BCa tissues have not been consistently reported [14-16]. However, the more recent study conducted by two independent medical institutes with 188 BCa and 141 normal bladder tissues showed that loss of ERα was commonly found in primary BCa tissues, and loss of ERα was strongly associated with higher grade and invasive tumors [14]. The difference between earlier reports [15-16] and the recent one [14] could possibly be attributed to improvements of antibody specificity and the methods and time periods of clinical specimen fixation [17, 18]. To date, there has been no study carefully evaluating ERα mRNA levels in a large number of clinical samples of BCa vs. non-cancerous specimens. With the above argument of IHC staining results [14-16], it is important to further validate the mRNA of ERα in clinical BCa specimens. In the present study, we analyzed 3 independent datasets to show reduced ERα mRNA in BCa compared to adjacent normal bladder tissues.

Currently, there have been reports of several mouse BCa models. These models include Uroplakin II promoter driven SV40 expression (UPII-SV40), N-butyl-N-(4-hydroxybutyl) nitrosamine (BBN)-induced, UPII-Ha-ras, etc [4, 19, 20]. Among these models, the pre-carcinogen BBN has been used extensively to induce BCa in mouse as reported in numerous publications [4, 21, 22]. BBN-induced BCa showed sexual dimorphism with males having a higher cancer incidence rate [4]. In addition, morphological characteristics of mouse BBN induced BCa are similar to those in humans [23, 24]. Thus, the BBN-induced mouse BCa model is widely applied as the best model to mimic human BCa development and was used for the present study.

Two types of categorized genes, proto-oncogenes and tumor-suppressor genes, play key roles in cancer induction. In bladder tumors, the proto-oncogene C-Met was positively associated with histologic grade, stage classification, and tumor size [25]. Pim-1, a proto-oncogene and serine/threonine-protein kinase, was expressed more in tumors than normal urothelial compartments and was higher in invasive BCa tissues [26]. Inositol polyphosphate-4-phosphatase, type II (INPP4B), a tumor suppressor, has a reduced expression in prostate and breast cancers compared to normal tissues [27, 28], yet its roles in BCa remain unclear. To understand ERα-mediated inhibition role in BCa, we screened a group of proto-oncogenes and tumor-suppressor genes, and found INPP4B was predominantly and specifically induced by ERα. Recently, INPP4B was shown to inhibit AKT phosphorylation by hydrolyzing phosphatidylinositol (3,4)-bisphosphate (PtdIns(3,4)P2) [29, 30]. Other than mechanistic studies, we have investigated and found that INPP4B expression, in correlation with reduced ERα, is reduced in human and mouse BCa tissues.

Together, we have utilized multiple *in vitro* and *in vivo* strategies to demonstrate ERα plays a protective role in BCa development and to investigate the functional mechanisms of ERα in BCa.

## RESULTS

### Reduced ERα mRNA in bladder cancers

**Figure 1:**
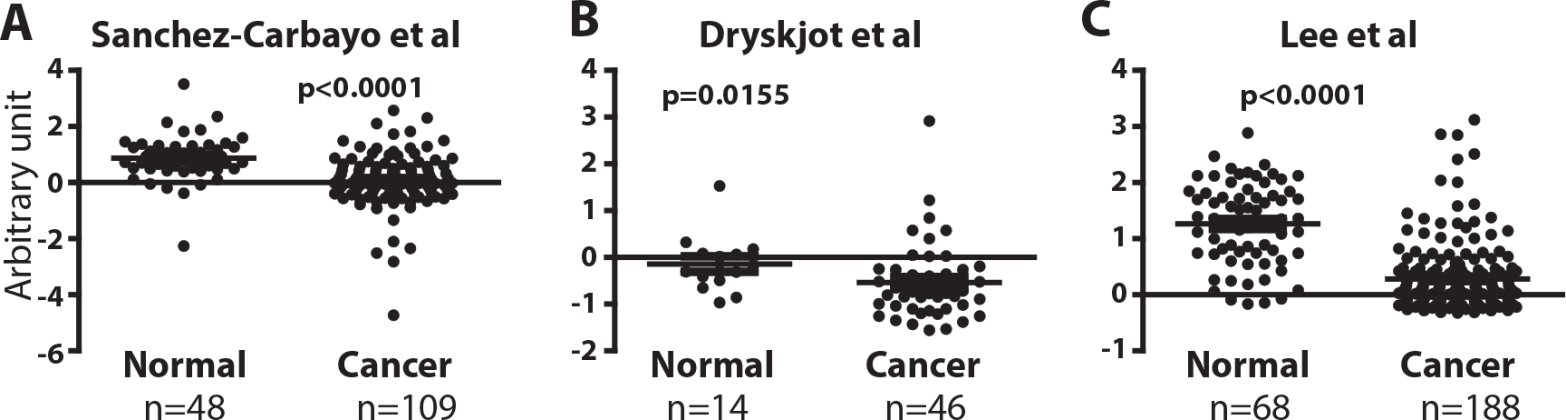
ERα mRNA expression is reduced in bladder tumors **(A, B, and C)** ERα expression was compared between normal bladder and BCa tissues in 3 independent databases by Sanchez-Carbayo et al. [33], Dyrskjot et al. [31], and Lee et al. [32]. Differences in distributions were tested by the *t*-test.

The roles of ERα in BCa development are under-studied. Currently, there are no available data concerning ERα mRNA level differences between normal and BCa tissue, and IHC staining results of ERα protein expression could be compromised by the antibody specificity, or the method and time of tissue fixation [17, 18]. Also, different from the IHC results that ERα protein is reduced in BCa [14], an earlier report of analyses of a small number of clinical specimen (10 cases) showed that ERα mRNA increases in BCa [16]. Thus, it was important to examine the ERα mRNA levels to compare with IHC results in larger numbers of clinical specimens. We analyzed mRNA expression results between normal and BCa tissues from the databases found in Oncomine (http://www.oncomine.org). In 3 separate microarray cohorts of bladder tissues (Study I: 48 normal tissues and 109 BCa tissues, Study II: 14 normal tissues and 46 BCa tissues, Study III: 68 normal tissues and 188 BCa tissues) [31-33], there were significantly lower mRNA expressions of ERα in tumors as compared with normal bladder tissues (Fig. 1). In conclusion, our report is the first comprehensive analysis of databases from 3 independent resources for ERα mRNA levels, and the analyzed data show that ERα mRNA is reduced in BCa. Together, Fig. 1 mRNA data and the reported IHC results [14] show ERα mRNA and protein decrease in BCa and ERα may play protective roles in BCa development.

### Knocking out ERα results in an increased cancer incidence in BBN-induced BCa model

**Figure 2:**
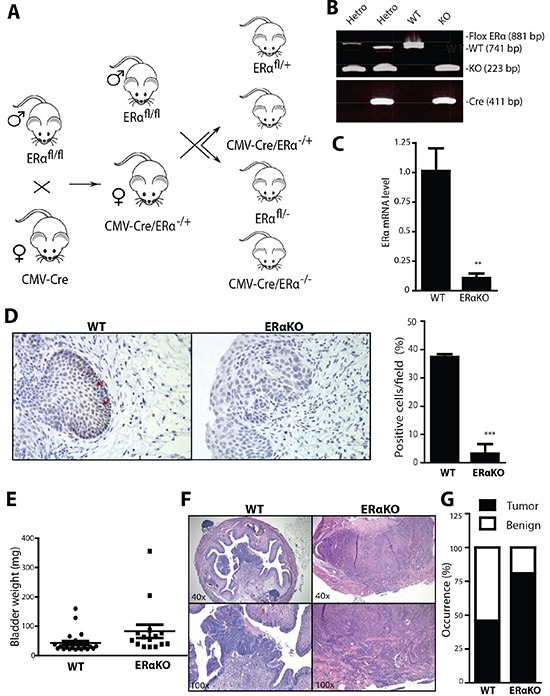
Total ERα knockout increased the cancer incidences in BBN-induced BCa model **(A)** ERαKO mouse breeding scheme. Female mice with Cre coding sequence under the control of human Cytomegalovirus promoter (CMV-Cre) were bred with male floxERα mice (ERα^fl/fl^) to generate heterozygous ERαKO mice (CMV-Cre/ERα^−/+^). Female CMV-Cre/ERα^−/+^ mice were then mated with male ERα^fl/fl^ mice to generate WT and ERαKO mice. **(B)** Tail genomic DNA was isolated and mouse genotypes were identified by PCR using primers flanking ERα exon 3 and Cre. **(C)** mRNA was collected from whole bladders of female WT and ERαKO mice. Quantitative PCR (qPCR) was used to compare ERα mRNA level. **, p<0.01 compared to WT mice by t-test. **(D)** ERα expression was detected in bladders of female WT and ERαKO mice by IHC. Red arrows indicate cells expressing ERα protein. Quantifications of ERα positive cells in the mice were shown at right (n=3 for each). ***, p<0.001 compared to WT mice by t-test. **(E)** Bladder weights were compared between WT (n=28) and ERαKO (n=16) female mice at 35 weeks old. p=0.0047 by t-test. **(F)** Representative images of BBN induced BCa of WT and ERαKO female mice at 35 weeks old. **(G)** BBN induced BCa incidence was compared between WT (n=28) and ERαKO (n=16) female mice at 35 weeks old. p=0.03 by Fisher's exact test.

To investigate the *in vivo* role of ERα in BCa development, we employed cre-loxP strategy to knock out the floxed ERα gene (ERαKO) [34-36]. The breeding scheme for the generation of CMV-ERαKO (Fig. 2A) is presented with CMV-Cre mice crossed with floxed ERα. Genotyping results showed that CMV-ERαKO mice have both Cre and ERαKO alleles (Fig. 2B). Quantitative-PCR results show ERα mRNA from bladders of CMV-ERαKO mice was barely detectable compared to WT mice (Fig. 2C). IHC staining results further confirmed that ERα protein expression is ablated in CMV-ERαKO bladders (Fig. 2D).

We utilized the pre-carcinogen BBN to induce BCa as a model to investigate ERα effect on cancer development. Mice reaching the age of 6 weeks were given BBN water for 12 weeks, and thereafter given regular water. Female mice were euthanized at 35-weeks-old and male mice at 30-weeks-old to determine the BCa incidence. Initial data analysis showed that ERαKO resulted in a higher bladder weight, an indicator of higher bladder cellularity and tumor mass [37] compared to WT bladders (Fig. 2E). Our data in Fig. 2F indicated ERαKO in female mice resulted in a higher BCa incidence of 81% compared to 46% in the WT mice (p<0.05, Fig. 2G). Histological analyses showed that these tumors mainly consist of transitional cell carcinomas, and include muscle invasive and non-muscle invasive tissues. Also, ERαKO males have a higher BCa incidence, yet the results did not reach a desired statistical difference (ERαKO 85% 11 out of 13 vs WT 63% 17 out of 27 mice, unpublished data). It is believed there may be other signaling pathways affecting BCa incidence in males, such as the androgen/AR pathway [4, 38]. Thus, a larger mouse sample size may be required in order to determine the male BCa rate with statistical significance. Overall, our data showed female CMV-ERαKO mice had a higher cancer incidence and tumor mass in the BBN-induced BCa model, suggesting ERα plays a protective role in female BCa formation.

**Figure 3:**
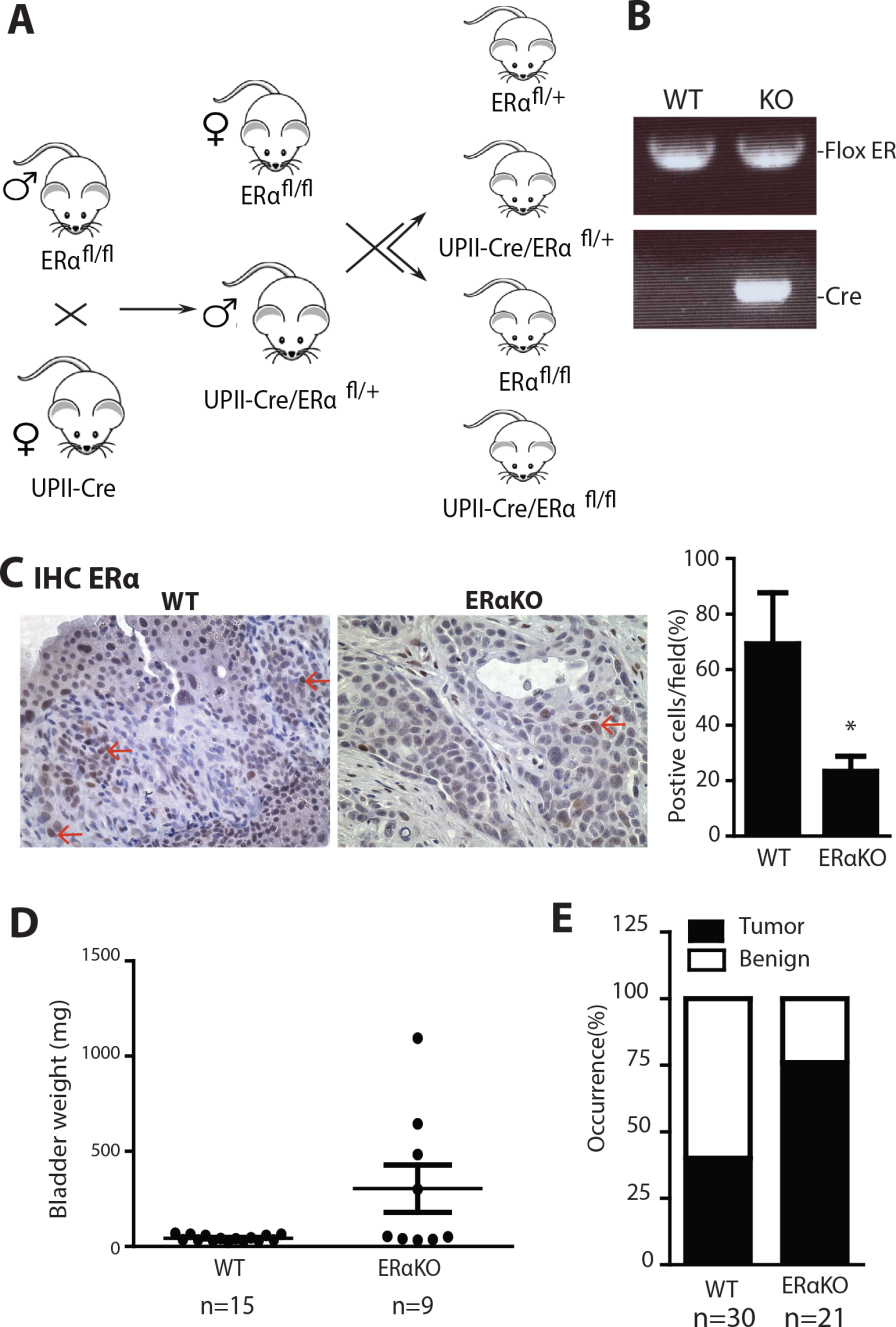
Urothelial specific ERα gene knockout increased the cancer incidences in BBN induced BCa model **(A)** Urothelial ERαKO mouse breeding scheme. Female mice with Cre coding sequence under the control of Uropleckin II promoter (UPII-Cre) were bred with male flox ERα mice (ERα^fl/fl^) to generate heterozygous ERαKO mice (UPII-Cre/ERα^fl/+^). Male UPII-Cre/ERα^fl/+^ mice were then mated with female ERαfl/fl mice to generate WT and UPII-Cre/ERαKO mice. **(B)** Tail genomic DNA was isolated for genotyping by PCR using primers flanking ERα exon 3 and Cre primers. **(C)** ERα protein expression was detected in female bladders of WT and ERαKO mice by immunohistochemistry. Red arrows indicate cells expressing ERα protein. Quantifications of ERα positive cells in the mouse bladder tissues were shown at right (n=4 for each) *, p<0.05 compared to WT mice by t-test. **(D)** Bladder weights were compared between WT (n=15) and UPII-ERαKO (n=9) female mice at 35 weeks old. p=0.0120 by t-test. **(E)** BBN induced BCa incidence was compared between WT (n=30) and UPII-ERαKO (n=21) female mice at 35 weeks old. p=0.0211 by Fisher's exact test.

In addition to CMV-ERαKO mice, we generated urothelium specific ERαKO mice (UPII-ERαKO) by breeding floxed ERα mice with UPII promoter driven Cre transgenic mice [39] (Fig. 3A). Genotyping results show that UPII-ERαKO mice have both Cre and floxed ERα alleles (Fig. 3B). The ERα KO allele cannot be detected from genotyping of DNA from tail snips of UPII-Cre driven knockout mice as Cre recombinase is only expressed in urothelial cells. IHC staining showed ERα protein is also ablated in UPII-ERαKO bladders (Fig. 3C). Female mice were fed with BBN water to induce tumors and then sacrificed at 35 weeks old. Higher bladder weight was found in the UPII-ERαKO mice than in WT mice (Fig. 3D) and histological analysis indicated that UPII-ERαKO mice have higher BCa incidence (76%) than WT mice (40%) (Fig. 3E), consistent with the phenotypes found in the CMV-ERαKO mice.

### ERα inhibits the malignant transformation of urothelial cells

**Figure 4:**
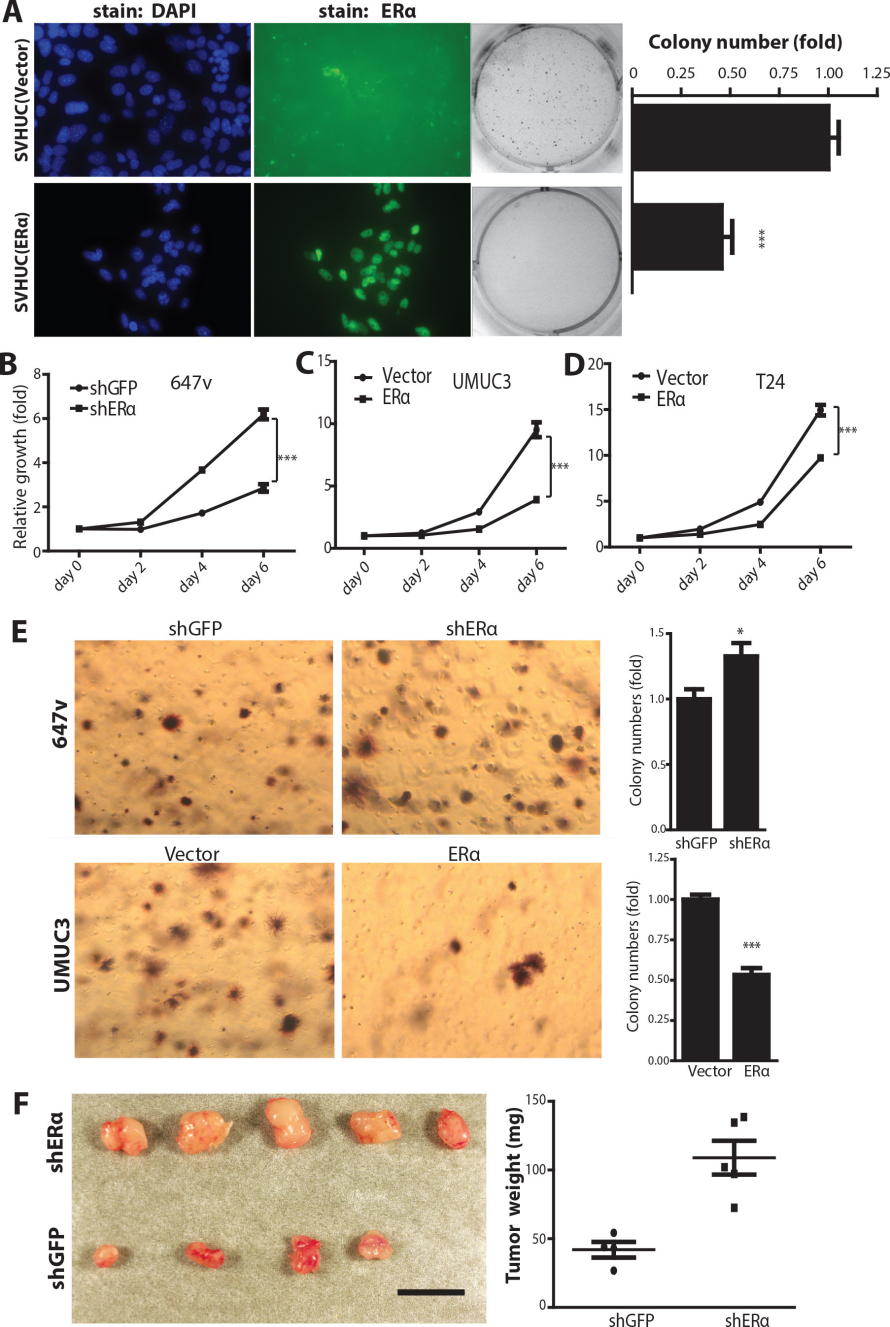
ERα inhibits carcinogen-induced cell malignant transformation and BCa cell growth **(A)** ERα expression was detected by antibody (middle 2 panels) and DAPI immunofluorescence staining was used to detect nuclei (left 2 panels) in SVHUC/ERα+ and SVHUC/vector cells. Non-malignant SVHUC cells, with or without ERα expression, were subjected to malignant transformation by MCA carcinogen treatment for three cycles and cultured for another six weeks. Soft agar assays (right panels) were performed to analyze cell transformation ability. Quantitative analyses (far right panel) of the colony numbers. ***, p<0.001 by unpaired *t*-test. **(B)** MTT assay used to analyze cell growth in 647v (shGFP or shERα), **(C)** UMUC3 (vector or ERα), and **(D)** T24 (vector or ERα) cells. ***, p<0.001 by Two-way ANOVA test. **(E)** Anchorage independent cell growth assay of BCa cells, infected with lentiviral shRNA against GFP or ERα in 647v cells, and lentoviral ERα or vector control in UMUC3 cells. *, p<0.05, ***, p<0.001 compared to control cells by t-test. **(F)** Knockdown of ERα increases tumor growth in the 647v xenograft mouse model. Nude mice were injected with 647v cancer cells expressing shRNA against ERα or control GFP in both flanks. Mice were sacrificed six weeks after cancer cell implantations. Mice injected with 647v cells with ERα shRNA showed increased tumor weights compared to shGFP control group (left panel). Quantification of tumor weights at right (p<0.01 by *t*-test).

In addition to the *in vivo* BCa model, we employed SVHUC, a non-malignant urothelial cell line, to study the ERα effect on carcinogen-induced malignant transformation. An earlier report used 3-methylcholanthrene (MCA) to induce malignant transformation of SVHUC cells [40], so we applied the same approach to determine whether expression of ERα can alter the cell transformation ability. SVHUC cells, an ERα negative cell line, were infected with lentiviral PWPI-vector control or PWPI-ERα. ERα expression was detected by immunofluoresence staining (Fig. 4A, left panels). We found that cells with ERα expression were less susceptible to MCA-induced malignant transformation than control cells as demonstrated by the soft agar anchorage-independent growth assay (Fig. 4A, middle and right panels). Together, results from our *in vivo* animal CMV- and UPII-ERαKO BCa models (Figs. 2 and 3) and *in vitro* malignant transformation tests (Fig. 4A) indicate that ERα reduces BCa incidence.

### ERα negatively regulates the BCa cell growth

To investigate the ERα effect on BCa cell growth, 647v, a BCa cell line with endogenous ERα, was infected with lentiviral-shRNA against ERα (shERα) or control shRNA against green fluorescent protein (shGFP). ERα was successfully knocked down as shown via examining ERα protein expression as well as its target genes' expression levels (Supplemental Fig. 1). The growth of ERα knockdown or control 647v cells were compared and results showed that ERα knockdown had a growth advantage over control cells (Fig. 4B), suggesting that expression of ERα in BCa cells inhibits their growth. Consistently, expression of ERα via the lentiviral delivery system (PWPI-ERα) in ERα negative BCa cell lines, UMUC3 and T24 (Fig. 4C and 4D), resulted in growth retardation compared to control cells, again confirming the role of ERα as a suppressor for tumor growth.

Anchorage independent cell growth is a hallmark of higher tumorigenecity or metastatic potential. Equal amounts of 647v cells with lentiviral shGFP or shERα transduction were suspended in 0.35% agar and grown for 2 weeks. Our data showed that lower colony numbers were found in cells with ERα expression (Fig. 4E upper panels). Consistently, results showed that UMUC3 cells transduced with PWPI-ERα had lower colony numbers compared to cells transduced with PWPI-vector (lower panels), indicating that expression of ERα reduced anchorage independent BCa cell growth.

To investigate the ERα effect on *in vivo* BCa growth and malignancy, 647v cells transduced with shERα or shGFP control were subcutaneously inoculated into nude mice. We found that tumors from 647v-shERα cells were bigger than those from 647v-shGFP control cells (Fig. 4F). This indicates that ERα plays an inhibitory role and BCa cells with reduced ERα are more malignant than BCa cells with higher ERα.

Together, our data demonstrate that ERα acts as a cell growth inhibitor and reduces cell malignancy as demonstrated in both ERα positive BCa cells with ERα knockdown, and in ERα negative BCa cells with ectopic ERα expression using *in vitro* and *in vivo* tumor growth assays.

### ERα selectively modulates INPP4B expression to control AKT pathway

**Figure 5:**
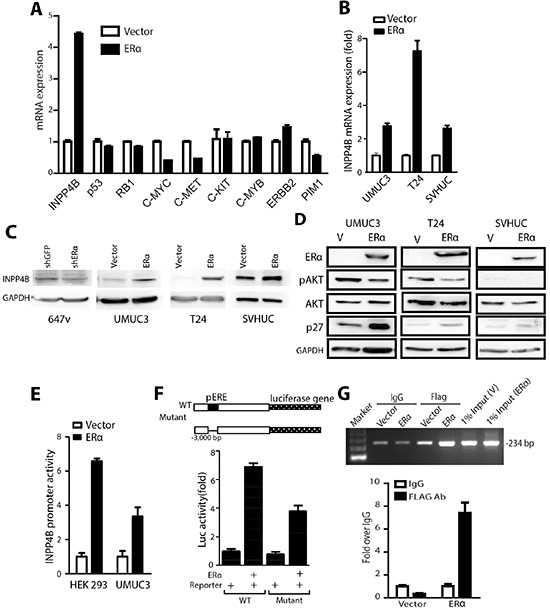
INPP4B expression is up-regulated by ERα in BCa cells **(A)** mRNAs were collected from UMUC3 cells transduced with PWPI-vector or PWPI-ERα. Q-PCR was performed to examine a panel of tumor suppressor or oncogene genes: INPP4B, p53, RB1, C-MYC, C-MET, C-KIT, C-MYB, BIRC2, and PIM1 expressions. Results were normalized to GAPDH expression. **(B)** mRNAs were collected from UMUC3, T24, and SVHUC cells transduced with PWPI-vector or PWPI-ERα. Expression levels of INPP4B were analyzed by qPCR. Results were normalized to GAPDH expression. **(C)** Protein lysates were collected from 647v cells transduced with shGFP or shERα or from UMUC3, T24, and SVHUC cells transduced with PWPI-vector or PWPI-ERα, followed by western blot detection of INPP4B and GAPDH protein expression. **(D)** Protein lysates were collected from UMUC3 cells (left panel), T24 cells (middle panel), and SVHUC cells (right panel) transduced with PWPI-vector or PWPI-ERα. Western blot was used to detect phosphorylated AKT, total AKT, p27, ERα, and GAPDH. **(E)** 3kb promoter of INPP4B was cloned to PGL3 firefly luciferase plasmid (INPP4B(3kb)-Luc) and transfected with pCDNA-ERα or vector, into HEK 293 and UMUC3 cells. **(F)** HEK 293 cells were transfected with WT or ERE deletion mutant INPP4B(3kb)-Luc and pCDNA3-ERα for 24 h, and cell lysates were assayed for luciferase activity. Transfection efficiency was normalized to renilla luciferase activity. **(G)** T24 cells with Vector or FLAG tagged ERα expression was used for CHIP assay. 234 bp was amplified from primers designed flanking the −2520 bp to −2287 bp region of INPP4B promoter. FLAG antibodies against FLAG tagged ERα and IgG were used as controls to pull down protein-DNA complex. 1% input was loaded as positive control. Amplified PCR products were quantified by qPCR and normalized to IgG control shown in lower panel.

Our data from clinical specimen analyses, animal models, and cell malignant transformation have consistently shown that ERα plays a protective role in BCa development. We were interested in understanding whether ERα regulates or cross-talks with oncogenes or tumor suppressor genes to mediate BCa development. We selected a panel of oncogene and tumor suppressor genes reported to be important in mediating cancer physiology, especially cell growth, to screen in ERα negative vs. positive cells. From the screening we found that ERα does not regulate p53, ERBB2, or C-MYB mRNA, but selectively controls the expression of INPP4B (Fig. 5A). Also, our results showed that higher ERα expression in BCa could reduce c-Myc and c-Met expression that may subsequently alter cell growth. The primary test of the change of c-Myc or c-Met by shRNA did not have prominent effect compared to the change of INPP4B to influence BCa growth (data not shown). Therefore, INPP4B was chosen as ERα downstream effector to be further characterized.

INPP4B was recently demonstrated to have reduced expression in cancer cells and was identified as a tumor suppressor in breast and prostate cancers [27, 28], yet its role in BCa remains unclear. Although an earlier report showed that INPP4B could be downstream of ERα in breast cancer, there is no functional linkage and no pathophysiological characterization of how ERα could regulate this pathway to impact any type of cancer development. Our quantitative data showed INPP4B mRNA was induced by ERα in BCa cells and non-malignant SVHUC cells (Fig. 5B). Consistent with the effect of INPP4B mRNA induction by ERα, the INPP4B protein level was also increased in cells with ERα (Fig. 5C).

As ERα is a well-known transcriptional factor [9, 41], we postulated the regulation of INPP4B mRNA is at the transcriptional level. To further dissect how ERα regulates INPP4B expression, 3kb of INPP4B gene promoter region was cloned into PGL3 Luciferase-reporter vector, INPP4B(3kb)-Luc, so that the promoter activity could be monitored by detecting firefly luciferase activity. HEK 293 cells were transfected with INPP4B(3kb)-Luc, vector or ERα expressing plasmids for 24 hr, and Luciferase activity results showed cells with ERα expression can upregulate INPP4B promoter activity around 6 fold compared to the control (Fig. 5E). ERα can also activate the INPP4B promoter activity in BCa UMUC3 cells by 3 fold (Fig. 5E), suggesting increased INPP4B mRNA by ERα is through transcriptional regulation. We then investigated the 3kb promoter and found there is a putative non-classic ERE site (located in -2353) that might be bound by ERα and act as an important element to control INPP4B expression. INPP4B-3kb-Luc with deletion of putative ERE was made (mutant INPP4B-3kb-Luc). Fig. 5F showed that the ERα activated INPP4B promoter activity is partially reduced in this mutant reporter, suggesting that ERα can partially regulate INPP4B promoter activity via this non-classic (-2353) ERE. This observation is supported by examining ERα effects on different lengths of the INPP4B promoter (Supplemental Fig. 2A). There is reduced luciferase activity in the 2kb length of INPP4B promoter compared to 3kb INPP4B promoter suggesting this ERE (located in -2353) could contribute to ERα regulated INPP4B promoter activity. Although no ERE within 2kb of INPP4B promoter was observed, there are C/EBP binding element that are reported to collaborate with ERα to activate gene expression [42]. Supportively, our chromatin immunoprecipitation (ChIP) assay results showed that ERα can bind to the -2520 to -2287 region of the INPP4B promoter where the putative ERE region is located (Fig. 5G and Supplemental Fig.7), suggesting ERα binds to the putative ERE region to regulate INPP4B expression. In addition, ChIP data further showed that ERα could bind to this putative C/EBP site (supplemental Fig. 2 and supplemental Fig.7), suggesting ERα could collaborate with other transcription factors for INPP4B promoter regulation.

It was reported that INPP4B could modulate AKT phosphorylation by hydrolyzing PtdIns(3,4)P2, which can associate with AKT and lead to AKT phosphorylation [29]. Thus, we examined whether ERα can affect AKT phosphorylation through changing INPP4B expression. Data showed that higher ERα expression reduces AKT phosphorylation at Ser 473, and also the expression of p27, a gene inhibited by AKT, was concomitantly induced by ERα (Fig. 5D). Although there are other genes downstream of AKT, our data have proved the regulatory pathway of ERα→induced INPP4B→inhibited AKT→increased p27→inhibited BCa cell growth.

### Knockdown of INPP4B reverses the ERα-inhibited urothelial malignant transformation and BCa growth

Decreased INPP4B expression has been reported to contribute to prostate and breast cancer development. To date, there is no report showing the connection of INPP4B with BCa development. We first tested whether INPP4B is a critical factor for the BCa growth. We introduced shRNA against INPP4B or control shRNA (shLuciferase) into BCa cells, J82 and T24, and examined whether the shRNA can knock down INPP4B and activate downstream effectors, such as AKT phosphorylation. We found that with INPP4B knockdown in the J82 and T24 cells, phosphorylation of AKT at Ser 473 can be increased and the BCa cells have a higher growth rate compared to control cells (Supplemental Fig. 2A and 2B).

**Figure 6:**
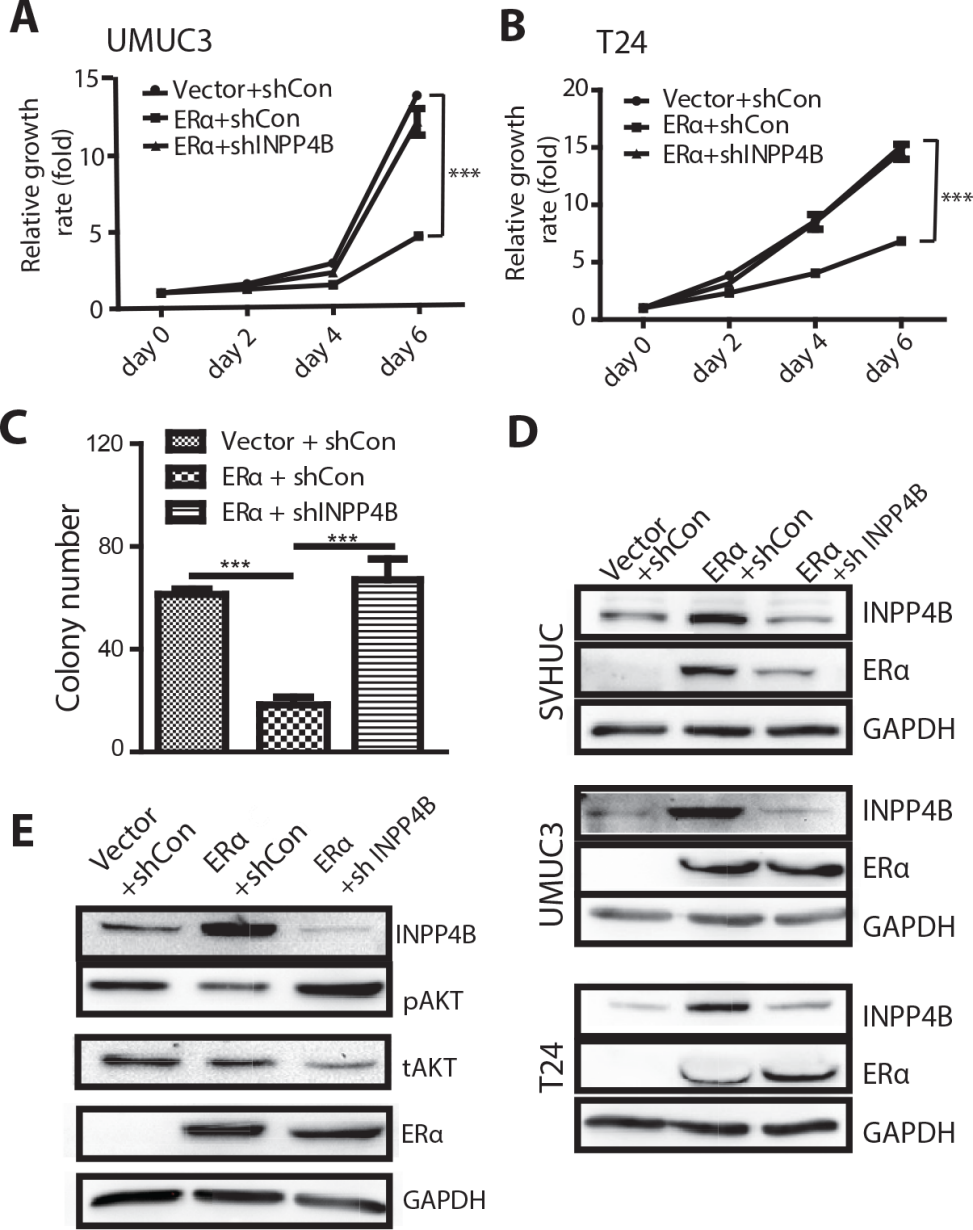
Knocking down INPP4B in BCa cells results in reversal of ERα inhibited BCa cell growth and ERα inhibited malignant transformation of SVHUC cells UMUC3 **(A)** and T24 **(B)** were transduced with lentiviral ERα and/or shINPP4B to investigate the functional connection of ERα and INPP4B in BCa cell growth. BCa cells were first infected with lentiviral ERα or vector (ERα(+), vec) and then further infected with lentiviral shINPP4B or sh control. We compared 3 groups of cells: (i) vector/shCon, (ii) ERα+/shCon, or (iii) ERα+/shINPP4B to assay cell growth on Days 0, 2, 4 and 6. ***, p<0.001 by Two-way ANOVA test. **(C)** SVHUC cells were transduced with lentiviral ERα and/or shINPP4b to investigate the functional connection of ERα and INPP4B in bladder cell malignant transformation. SVHUC cells with vector/shCon, ERα(+)/shCon, or ERα(+)/shINPP4B transduction were subjected to MCA carcinogen treatment. Soft agar assays were performed to analyze malignant transformation ability. Quantitative analysis of the colony numbers of the soft agar assays is shown. ***, p<0.001 by one way ANOVA test. **(D)** Protein lysates were collected from SVHUC, UMUC3, and T24 cells with lentiviral vector/shCon, ERα(+)/shCon, and ERα(+)/shINPP4B transduction. Immunoblotting was performed with antibodies against ERα, INPP4B, and GAPDH. **(E)** Protein lysates were collected from UMUC3 cells with lentiviral vector/shCon, ERα(+)/shCon, and ERα(+)/shINPP4B transduction. Immunoblotting was performed with antibodies against ERα, INPP4B, phosphorylated AKT (pAKT), total AKT (tAKT), and GAPDH.

As INPP4B is the direct downstream effector of ERα functions, we were interested in testing whether knockdown of INPP4B can reverse ERα mediated growth inhibition in BCa. We introduced shRNA against INPP4B into ERα expressing BCa cells. The results first showed that ERα positivity could reduce BCa cell growth compared to cells not expressing ERα (Fig. 6A and B). When INPP4B was knocked down in the T24 ERα(+) and UMUC3 ERα(+) BCa cells, the ERα mediated cell growth reduction was abolished (Fig. 6A and 6B), suggesting INPP4B knockdown can reverse ERα mediated inhibition effect on BCa cell growth. Because INPP4B has a baseline expression in the ERα(-) BCa cells, it is not surprising to see that INPP4B knockdown in the ERα(-) BCa cells also showed an increased cell growth (Supplemental Fig. 2C).

Furthermore, we tested whether INPP4B knockdown may reverse ERα controlled and inhibited malignant transformation of SVHUC cells. First, ERα expression reduced malignant transformation ability as is reflected by a lower colony number in the soft agar assay, as compared to ERα(-) SVHUC cells. With INPP4B knockdown in ERα(+) SVHUC cells, the colony number was increased to a similar level as ERα(-) cells (Fig. 6C), suggesting that knockdown of INPP4B in the non-malignant urothelial cells can reverse ERα inhibited malignant transformation. The protein expression levels for INPP4B and ERα in different cells are shown (Fig. 6D).

Importantly and supportively, the ERα inhibited AKT phosphorylation also recovered following INPP4B knockdown, indicating INPP4B is an important ERα downstream effector to regulate AKT phosphorylation (Fig. 6E).

To test whether changes in AKT activation status correlates with the cause of ERα inhibited cell growth, we examined ERα growth effects on cells with ectopically expressed constitutive active AKT (Supplemental Fig. 4). Data showed that ERα–mediated growth inhibition effect can be partially reversed by ectopic expressed constitutive AKT (Lane 4 vs lane 2), suggesting changes of AKT activation status is one of the factors contributing to ERα growth inhibition effect, however, other unidentified factors could be involved and need to be characterized.

### Reduced INPP4B expression and increased AKT activity in ERαKO mouse BCa model

**Figure 7:**
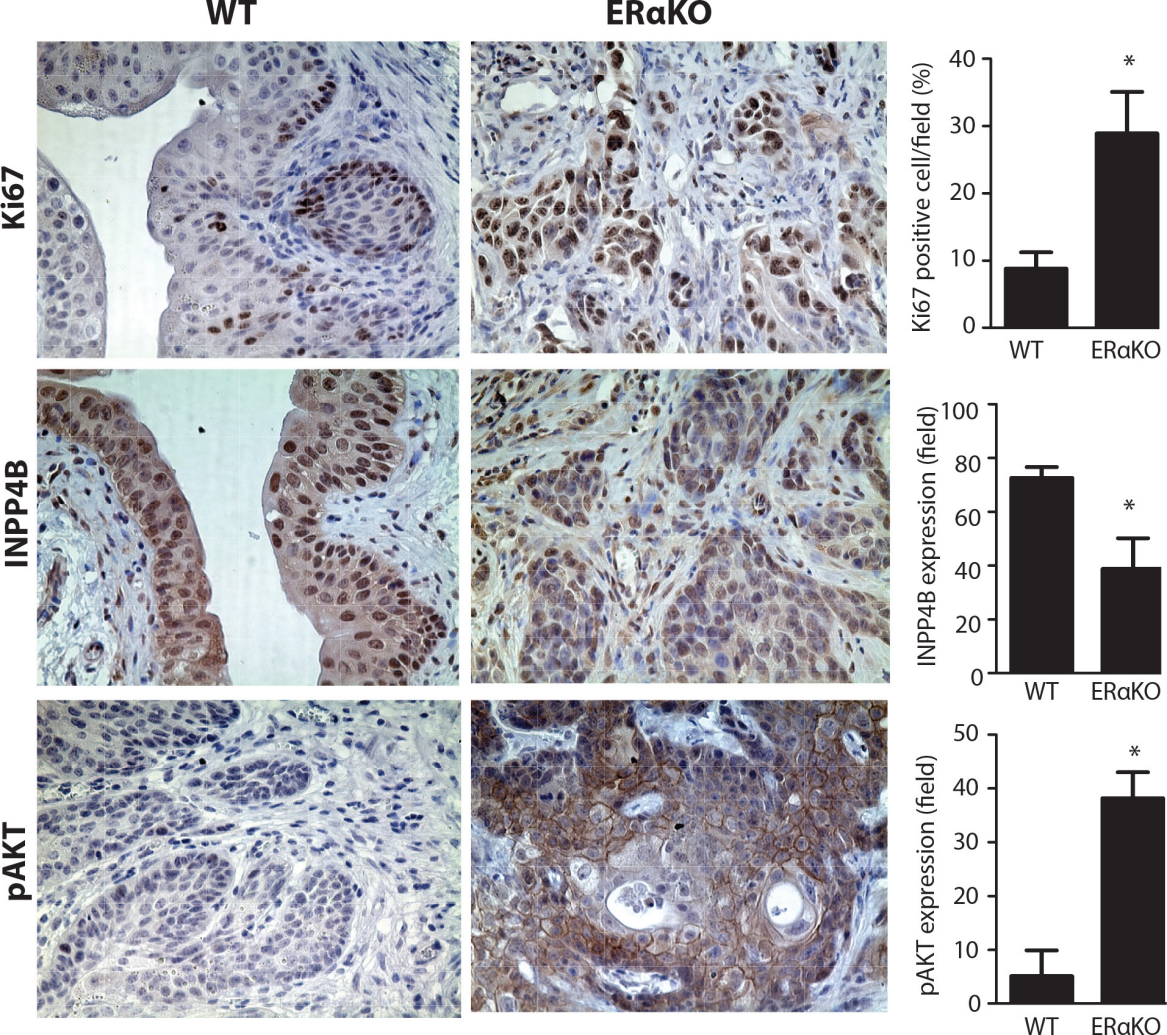
Reduced INPP4B and pAKT expression in BBN treated mouse BCa tissues Immunohistochemical staining was performed and compared in BBN induced mouse BCa from WT and CMV-ERαKO female mice. IHC was performed with antibodies against Ki67 (upper panels), INPP4B (middle panels), and pAKT (lower panels) (n=3 for each group), in the bladder tissues from BBN treated WT and CMV-ERαKO female mice at 35 weeks old. *, p<0.05 compared to WT by t-test.

In addition to validating the ERα→increased INPP4B→decreased AKT mechanism in BCa cells and non-malignant cells, we investigated whether ERα expression can regulate INPP4B expression and AKT activity in the *in vivo* mouse model. We collected bladder tissues from BBN treated WT and ERαKO female mice at 35 weeks and detected INPP4B protein and AKT phosphorylation. Ki67, a proliferation marker, was also detected to indicate proliferating cells (Fig. 7, upper panels), and our results indicated cells from ERαKO mice are highly proliferative as indicated by a high Ki67 staining amount. Importantly, INPP4B protein showed reduced expression in ERαKO BCa compared to WT BCa tissues (Fig. 7 middle panels and Supplemental Fig. 4). Concomitantly, AKT activity was induced in ERαKO BCa compared to WT BCa tissues, indicating ablation of ERα can enhance AKT activity and this effect could be through the reduced INPP4B (Fig. 7, lower panels).

### Consistent with reduced ERα, INPP4B expression is reduced in human BCa specimens

**Figure 8:**
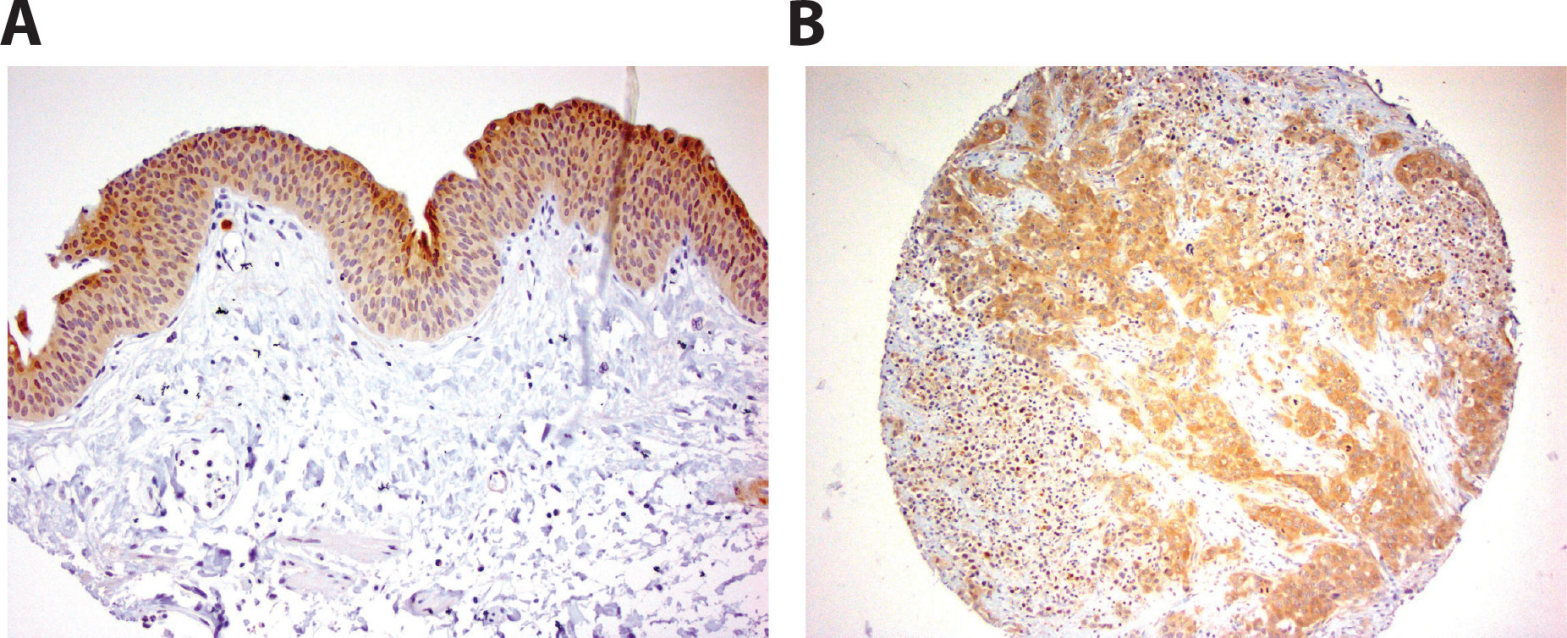
INPP4B immunoreactivity in human bladder tissue Strong staining in benign urothelium **(A)** and urothelial tumor **(B)** is detected. Original magnification in (A) and (B) is x200.

We investigated the expression of INPP4B in 129 bladder tumor specimens as well as 87 benign bladder tissues by immunohistochemistry staining. Positive signals were detected predominantly in the cytoplasm of benign/malignant epithelial cells (Fig. 8). Correlations of the expression status with different non-neoplastic and neoplastic bladder tissues are summarized in Table 1. INPP4B was positive in 76 of 87 (87%; 38: 1+, 32: 2+, and 6: 3+) benign urothelial tissues and 80 of 129 (62%; 51: 1+, 23: 2+, and 6: 3+) primary tumors. Overall, INPP4B expression was significantly lower in tumors than in benign urothelium (P<0.001).

**Table 1:** Expression of INPP4B in bladder tissue microarrays

INPP4B expression						P value		
	N	0	1+	2+	3+	0 vs 1+/2+/3+	0/1+ vs 2+/3+	0/1+/2+ vs 3+
**Benign**	87	11 (12.6%)	38 (43.7%)	32 (36.8%)	6 (6.9%)	<0.001	0.001	0.551
**Tumor**	129	49 (38.0%)	51 (39.5%)	23 (17.8%)	6 (4.7%)			
								
**Low-grade**	52	13 (25.0%)	25 (48.1%)	13 (25.0%)	1 (1.9%)	0.016	0.391	0.400
**High-grade**	77	36 (46.8%)	26 (33.8%)	10 (130.%)	5 (6.5%)			
								
**pTa-1**	77	20 (26.0%)	34 (44.2%)	19 (24.7%)	4 (5.2%)	<0.001	0.018	1.000
**pT2-4**	52	29 (55.8%)	17 (32.7%)	4 (7.7%)	2 (3.8%)			
								
**ERα(−)**	95	41 (43.2%)	38 (40.0%)	13 (13.7%)	3 (3.2%)	0.063	0.008	0.340
**ERα(+)**	34	8 (23.5%)	13 (38.2%)	10 (29.4%)	3 (8.8%)			

We next evaluated the correlation of INPP4B expression with clinicopathologic profiles available for our patient cohort. There was no significant difference in INPP4B expression pattern between male vs. female tumors. Thirty-nine out of 52 (75%) low-grade tumors were INPP4B positive, whereas 41 out of 77 (53%) high grade carcinomas were INPP4B-positive (P=0.016). Similarly, 57 of 77 (74%) non muscle invasive tumors expressed INPP4B, compared with 23 of 52 (44%) muscle invasive tumors (P<0.001). There were also correlations between statuses of ERα and INPP4B in tumors (0/1+ vs. 2+/3+, P=0.008).

Although in one earlier report, INPP4B could be co-localized with ERα in luminal cells of breast cancer, yet there was no functional linkage and no pathophysiological characterization how ERα could regulate this INPP4B pathway to impact any type of cancer development. Especially, INPP4B suppressor function is in contrast with ERα tumor promoting roles in breast cancer. Our work is the first report to show pathophysiological characterization of how ERα interplays with INPP4B to inhibit bladder malignant transformation and cancer growth *in vivo* as well as *in vitro*.

Together, we provide evidence from multiple *in vitro* cell studies and *in vivo* cre-loxP ERαKO tumor models, as well as human BCa tissue data to prove ERα plays a protective role in BCa initiation and growth via modulating the INPP4B/Akt pathway.

## DISCUSSION

### Early studies about estrogen effects in BCa development

Earlier reports showed that ERα promotes cancer development in breast, ovarian and endometrium [43-46]. In contrast, ERα inhibition of cancer incidence could be observed in liver and colon cancers, where males have higher cancer incidence than females [47-49]. How estrogen and ERs regulate BCa development remains to be further characterized. There has been a report using synthetic estrogen or 17 β-estradiol (E2) to treat male rats in BBN induced BCa models, and results showed that male rats receiving estrogen have lower BCa incidence compared to vehicle treated rats [50]. Although the results suggested that rats with estrogen exposure have a reduced BCa incidence, some concerns were raised from these studies. First, the supplementation of estrogen to the rats was not within physiological range. Second, the super physiological doses of estrogen could inhibit the production of testicular androgens in male rats. In addition, other hormone levels including progesterone, Follicle-stimulating hormone and Luteinizing hormone, could be dramatically altered in these estrogen over-exposed animals. Other prior reports also showed controversial roles of ERs in BCa growth [16, 51].

### Other supportive reports as well as our evidences about the protective roles of ERα in BCa

Therefore, in order to specifically characterize ERα effect on BCa development without the compromised phenotypes from altered hormonal profiles, the mouse BCa models with cre-loxP ERα gene knockout (CMV-ERαKO and UPII-ERαKO) were chosen for our study. In this study, we provide evidence from *in vitro* cell lines studies and *in vivo* mouse models to demonstrate that ERα inhibited BCa incidence and growth. In addition to our results, other studies also imply that reduced ERα signaling may be associated with BCa incidence. For example, arsenic exposure and schistosomiasis infections are linked to increased BCa risk and both events have been associated with reduced ERα signaling [52, 53]. Furthermore, people who carry the *ERα*-*397 T* allele*,* have a higher susceptibility to BCa [54], implying ERα signaling is associated with BCa incidence. Together, ERα inhibition effects on both malignant transformation and BCa cell growth indicate that urothelium ERα expression plays protective roles against BCa development, consistent with *in vivo* mouse data in which ERαKO mice have a higher BCa incidence. Together, there are other supportive reports as well as our data demonstrating the protective roles of ERα in BCa.

### Differential roles of ERα and ERβ in BCa

The sexual difference of BCa occurrence suggests that estrogen and its receptors may play roles in BCa development. However, there are two major types of estrogen receptors, ERα and ERβ, to mediate estrogen effects. Treatments with selective ER modulators, including raloxifene and tamoxifen, have been shown to reduce BCa growth [55]. Our recent results with cre-loxP ERβKO show inhibition of mouse BCa development, suggesting ERβ promotes BCa development. Mechanism dissection found that targeting ERβ suppressed the expression of minichromosome maintenance complex component 5 (MCM5), a DNA replication licensing factor that is involved in tumor cell growth. Restoring MCM5 expression can partially reverse ERβ knockdown-mediated growth reduction. Supportively, treating cells with the ERβ-specific antagonist, 4-[2-Phenyl-5,7-bis(trifluoromethyl) pyrazolo[1,5-a]pyrimidin-3-yl]phenol (PHTPP), reduced BCa cell growth and invasion, as well as MCM5 expression [56]. The present study showed that ERα plays a protective role in BCa via upregulation of INPP4B and inhibition of Akt. In addition, human BCa tissue ERα and ERβ staining results conclude that loss of ERα was strongly associated with higher grade/more invasive tumours, whereas ERβ expression was increased in high-grade/invasive tumours and its presence predicted a worse prognosis [14]. Together, our data support the concept that ERα and ERß could play differential roles in BCa development [56, 57].

### ERα expression is reduced in cancer tissues

We observed less ERα mRNA level in the cancer tissues from various stages compared to normal tissues either from histologically normal-looking surrounding or normal bladder tissues. Although the magnitude of change between the non-malignant and BCa is not huge, it is still meaningful due to statistical significance and considering the potential variation of human tissue analysis. Because of this observation and the BCa incidence difference of male to female (3.5:1), we hypothesized ERα may play a protective role in BCa initiation and examined whether altered ERα expression can influence BCa development using *in vitro* cell studies and *in vivo* animal models. The mechanism studies showed that ERα could function via regulating of INPP4B/inhibited Akt pathway to control BCa development.

Among 3 data sets we analyzed, we observed consistent ERα mRNA reduction in BCa. However, there is no consistent change of INPP4B mRNA among these 3 BCa databases. It is not surprisingly to see the somewhat different clinical data analyses, which may involve the different platforms used (Affymetrix or Illumina), probes designed, or tissue preparation (biopsies vs. whole tissues) [58]. Importantly and notably, INPP4B has been demonstrated as a tumor suppressor in different cancer types such as prostate and breast [27, 30, 59], consistent with what we have observed for BCa using IHC staining of the INPP4B protein (Fig. 8). The change of INPP4B mRNA in BCa tissues merits more investigations. Overall, we observed the correlated ERα and INPP4B reduction at protein levels.

### Estrogen exposure inhibits bladder cancer incidence and management of ERα signal pathway may be a potential agent for preventing bladder cancer

There are also reports suggesting that estrogen inhibits BCa incidence. Postmenopausal women have a higher risk of BCa than premenopausal women [60], and women who reach menopause at a younger age have a significantly increased risk of BCa [61-63], supporting the concept that estrogens might inhibit BCa incidence. Additional clinical studies also supported that higher frequencies of estrogen exposure might lead to less BCa incidence. For example, parous women (during pregnancy, estradiol increases) [61-65] and those who used estrogen and progestin for hormonal therapy [63, 64] have a lower risk for BCa formation, again suggesting high estrogen exposure decreases BCa risk. These observations are consistent with what we found that estrogen can enhance ERα effect on inhibiting BCa growth (Supplemental Fig. 6A). The concept is further supported with the observation that ERα mutants (Y537S and D538G), which have mutations in the ligand binding domain and have been demonstrated to maintain transcriptional activity [66, 67] in the absence of estrogen, showed dramatic growth inhibition effect on BCa cells (Supplemental Fig. 6B). Those results imply that higher frequency of estrogen exposure may protect women from BCa occurrence, suggesting estrogens play protective roles in BCa initiation and might serve as therapeutic agents.

### Potential applications of Akt inhibitors, ERα selective agonist and downstream pathways for BCa therapy

Our studies proved that ERα can up-regulate INPP4B expression and concomitantly reduce AKT phosphorylation status and activity. An earlier report showed AKT activity is increased in BCa and has been correlated to BCa stage [68]. Furthermore, inhibitors against AKT activity have been developed for use in pre-clinical trials [69]. The effect of AKT inhibitors on cell growth was also evaluated in a variety of cancers [70, 71]. Our ERα-INPP4B mechanism results support that AKT inhibitors may be applied to treat BCa. In addition, our data proved that ERα inhibits cell transformation and cancer cell growth. It is possible that propylpyrazoletriol (PPT), a selective agonist for ERα, may be applied to treat BCa patients and to inhibit BCa cells growth. From the screening to examine which oncogenes or tumor suppressors can be altered by ERα, we found that ERα expression in BCa can reduce c-Myc and c-Met expression, which could subsequently alter cell growth. This might provide an alternative pathway for ERα to regulate cell growth.

Together, our study has provided multiple evidences to demonstrate ERα inhibition role in BCa development both *in vitro* and *in vivo* by modulating the INPP4B/Akt pathway, and suggest that the ERα agonist, PPT, or AKT inhibitors could be used as therapeutic agents in the future to control BCa development.

## MATERIALS AND METHODS

### Generation and genotyping of CMV-Cre/ERα−/− (CMV-ERαKO) or UPII-Cre/ERα^fl/fl^ (UPII-ERαKO) mice

The ERα^fl/fl^ mouse with lox sequence flanking exon 3 of ERα allele (floxed ERα) was produced as previously described [36, 72]. CMV-ERαKO mice were generated by crossing ERα^fl/fl^ male mice with CMV-Cre transgenic female mice (Jackson Laboratories, Bar Harbor, ME). UPII-ERαKO mice were generated by crossing ERα^fl/fl^ female mice with UPII-Cre transgenic male mice (obtained from Dr. Xue-Ru Wu, NYU). The sizes of the wild type ERα allele, floxed ERα allele, and ERα KO allele were 741, 881, and 223 bps, respectively. To identify Cre recombinase bearing transgenic mice, primer sequences located in the Cre coding regions were used. The PCR product of the Cre fragment was 411 bps. ERα KO allele can be detected from genotyping of DNA from tail snip in CMV-Cre driven knockout but cannot be detected in UPII-Cre driven knockout.

### Bladder Samples for ESR1 mRNA analysis

In Dyrskjot bladder studies, there are 14 normal biopsies and 46 cancer samples where cancer tissues are biopsies from 28 superficial bladder tumors (Ta and T1) (13 tumors with surrounding CIS and 15 without surrounding CIS) and 13 invasive carcinoma (T2 to T4) and biopsies from 5 CIS patients. In Lee bladder studies, there are 68 normal and 188 cancer tissues. Normal tissues are from fifty-eight samples of histologically normal-looking surrounding tissues from the patients with urothelial carcinoma and 10 normal bladder mucosae from patients with benign diseases. Cancer tissues are from 126 samples of superficial bladder tumors (Ta and T1, recurrent and not recurrent) and 62 invasive carcinoma (T2 to T4). In Sanchez-Carbayo Bladder studies, there are 48 normal urothelium and 109 cancer samples. For cancer tissues, 28 samples of superficial bladder tumors (Ta-Tis-T1) and 81 invasive carcinoma tissues (T2-T4).

### Inducing BCa with BBN in drinking water

Both WT and ERαKO female mice in C57BL/6 background were supplied with sterile distilled water containing 0.05% BBN (TCI America) at 6 weeks old for 12 weeks and thereafter with normal drinking water until the mice were sacrificed [4].

### Immunoblotting and Immunohistochemical staining (IHC)

BCa cells were lysed for Western blotting and proteins were detected using the antibodies, GAPDH (Santa Cruz 32233), P-Ser473 AKT (Cell Signaling 4060), INPP4B (Santa Cruz 12318), Total AKT (Cell Signaling 9272), ERα (produced in our lab, SC1-1), and p27 (Santa Cruz 528). Tissue fixation and sectioning were processed as previously described [36, 73]. Tissue sections were incubated with ERα antibodies (Santa Cruz, MC-20), P-Ser473 AKT (Cell Signaling 4060), INPP4B (Abcam EPR3108), Ki67 (Novocastra).

### RNA isolation and real time PCR

Detailed RNA isolation and real time PCR was as previously described [36, 72].

### Cell Culture and anchorage independent growth assay

UMUC3, 647v, and T24 cells were purchased from ATCC and cultured in DMEM with 10% fetal bovine serum. SVHUC cells were purchased from ATCC and maintained in F12-K with 10% fetal bovine serum. Anchorage independent cell growth was performed by plating 1% soft agar on the bottom of plates, allowing the agar to solidify and then seeding the suspending cells in 0.35% of soft agar in DMEM. Cells were grown in soft agar for two weeks and stained with 0.2 μg/ml Iodonitrotetrazolium chloride (Sigma-Aldrich) for 20 hr to indicate live cells. Live colonies were counted and compared between cells with and without ERα expression.

### Malignant transformation assay

Cell transformation protocol was followed according to Reznikoff *et al.* [40]. Briefly, 1×10^5^ of SVHUC cells cultured in 10-cm dishes were exposed to 5 μg/ml 3-methylcholanthrene (MCA) for 48 hr and then cultured for about 10 days until cells reached confluence followed by subculturing cells using a 1/3 split. The treatment cycle was repeated two more times. The recovered cells were cultured for 6 weeks by regular passage, and 2×10^5^ cells were used for soft agar assay to determine malignant transformation.

### Lentiviral vectors construction and virus production

The cDNA encoding ERα was cloned into PWPI lentiviral vector that was constructed with SV40-puro for cell selection purpose (PWPI-ERα). The short hairpin RNA (shRNA) expressing lentiviral vector against GFP (PLKO.1-puro-shGFP) and PLKO.1 hygro vector were obtained from Addgene. The shRNA against ERα (PLKO.1-puro-shERα) and control (PLKO.l-hygro-INPP4B scramble) and INPP4B (PLKO.l-hygro-INPP4B) were constructed with target sequence according to Addgene's pLKO.1 protocol. The target sequence for ERα was 5′-GTACCAATGACAAGGGAAGT-3′, for scramble INPP4B was 5′-GAATTATACCGTCAACTCTAA-3′ and for INPP4B was 5′-CCCTTCACATTAAAGAAGATT-3′. Lentiviral particles were generated and transduced into cells.

### INPP4B (3kb)-Luciferase and ERE mutant INPP4B(3kb)-Luciferase plasmid construction

A 3000-base pair fragment of the 5′-flanking region of the INPP4B gene was amplified by PCR from T24 genomic DNA and ligated into pGL3-Basic Vector (Promega, WI), designed as INPP4B(3kb)-Luc. The region is defined as promoter from Ensembl project [74]. The oligonucleotides used for PCR is 5′- CCGGGCTAGCATGACTGGGGGAAGACAAAAG-3′ (−3000 to −2987) with NheI restriction enzyme site and 5′-CCGGCTCGAGCAGGTGCCACCTGGCGGCTCTCT -3′ (−1 to −12) with XhoI restriction enzyme site. The ERE mutant INPP4B(3kb)-Luc has same promoter region with deletion from −2353 to −2366 bp.

### Luciferase reporter assay

Cells were transfected with empty vector (pcDNA3) or ERα expressing vector (pcDNA3-ERα) and PGL3-3kb-INPP4B promoter driven luciferase expressing vector and SV40 driven renilla expressing vector as control for 24 hrs followed by lysing cells with lysis buffer and assayed according to manufacturer's instructions (Dual-Luciferase Reporter Assay System, Promega).

### Immunohistochemical staining of INPP4B in human bladder tissue microarray

We retrieved 129 bladder specimens obtained from transurethral resections or cystectomy performed at the Johns Hopkins Hospital. All the sections were reviewed for confirmation of original diagnoses, according to the 2004 World Health Organization/International Society of Urological Pathology classification system for urothelial neoplasms [75], by an urologic pathologist (G.J.N.). Appropriate approval from the Institutional Review Board was obtained prior to construction and use of the tissue microarray (TMA). Bladder TMAs were constructed from formalin fixed paraffin embedded specimens (129 tumor tissues and 87 benign appearing tissues from bladders of patients with tumors), as previously described [14]. These patients included 98 men and 31 women, with a mean age of 65.7 years (range: 26-89 years) at the time of surgery and a mean follow-up of 32.7 months (range: 2-164 months) post surgery. The primary tumors included 12 papillary urothelial neoplasms of low malignant potential (PUNLMPs), 40 non-invasive (pTa) low-grade urothelial carcinomas, 26 non-muscle-invasive (≤pT1) high-grade urothelial carcinomas, and 51 muscle-invasive (≥pT2) high-grade urothelial carcinomas. All 51 patients with muscle-invasive tumors underwent cystectomy. None of the patients had received therapy with radiation or anticancer drugs pre-operatively, except for 14 cases with intravesical bacillus Calmette-Guérin treatment prior to radical cystectomy. All of these 129 cases were included in our prior study analyzing 188 cases for the expression of ERα [14].

Immunohistochemical staining was performed, using the primary antibody to INPP4B (HPA037682, 1:150 dilution, Sigma-Aldrich). All the stains were manually scored by an experienced and certified pathologist (H.M.) blinded to patient identity. The German Immunoreactive Score was calculated by multiplying the percentage of immunoreactive cells (0% = 0; 1-10% = 1; 11-50% = 2; 51-80% = 3; 81-100% = 4) by staining intensity (negative = 0; weak = 1; moderate = 2; strong = 3). The immunohistochemical scores (ranging from 0-12) were considered negative (0; 0-1), weakly positive (1+; 2-4), moderately positive (2+; 6-8), and strongly positive (3+; 9-12) for INPP4B expression. The Fisher's exact test was used to evaluate the association between categorized variables.

### Statistics

Differences in cancer incidence between BBN treated WT and ERαKO mouse studies were analyzed by Fisher's exact test. Student's t-test was used to test the differences of mRNA level and colony formation ability between lentiviral vector and lentiviral ERα transduced cells.

## SUPPLEMENTARY FIGURES AND TABLE



## Abbreviations

ERα: estrogen receptor alpha
ERαKO: ERα knockout mice
BCa: bladder cancer
BBN: N-butyl-N-(4-hydroxybutyl) nitrosamine
IHC: immunohistochemistry
INPP4B: Inositol polyphosphate-4-phosphatase type II
MTT: 3-(4,5-Dimethylthiazol-2-yl)-2,5-Diphenyltetrazolium Bromide
MCA: 3-methylcholanthrene
WT: wild type
