# Supplementary material for: Estrogen Receptor Alpha Prevents Bladder Cancer Development via INPP4B inhibited Akt Pathway in vitro and in vivo
Source: Oncotarget. 2014 Aug 13;5(17):7917–35. doi: 10.18632/oncotarget.1421 (PMC4202170; doi:10.18632/oncotarget.1421)
Supplement: Supplementary file 1 [file oncotarget-05-7917-s001.pdf]

SUPPLEMENTARY FIGURES AND TABLE

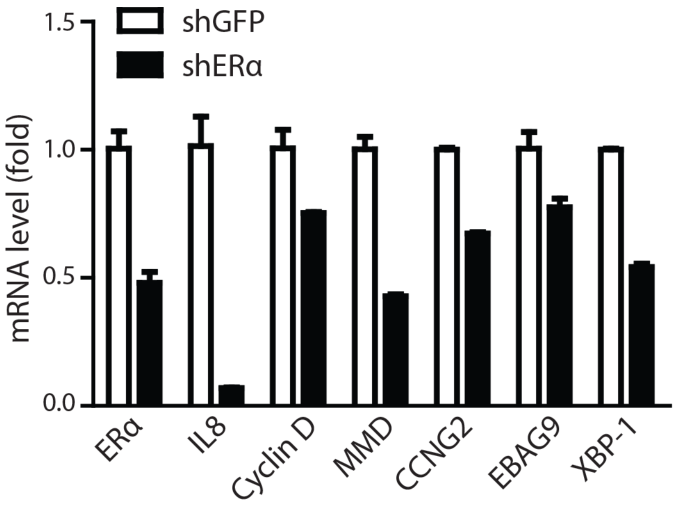

Supplemental Figure S1: Genes regulated by ERα in 647v. mRNAs were collected from 647v cells transduced with PLKO.1 shGFP or PLKO.1 shERα. Expression levels of ERα and ERα target genes including IL8, Cyclin D1, MMD, CCNG2, EBAG9, XBP-1 and GAPDH were analyzed by qPCR. Results were shown normalized to GAPDH expression.

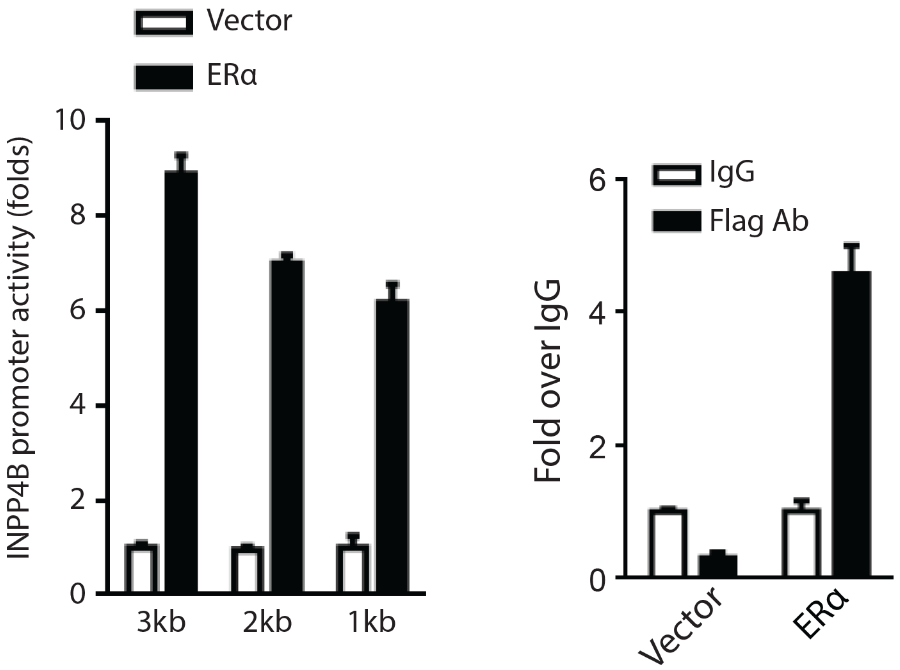

Supplemental Figure S2: INPP4B promoter is regulated by ERα and C/EBP transcriptional factor. (A) HEK 293 cells were transfected with INPP4B(3kb)-Luc, INPP4B(2kb)-Luc, INPP4B(1kb)-Luc, and pCDNA vector or pCDNA3-ERα for 24 h. Cell lysates were collected and assayed for luciferase activity. Transfection efficiency was normalized to renilla luciferase activity. **(B)** T24 cells with Vector or FLAG tagged ERα expression was used for CHIP assay. 158 bp was amplified from primers designed flanking the -901 to -1058 bp region of INPP4B promoter. FLAG antibodies against FLAG tagged ERα and IgG were used as controls to pull down protein-DNA complex. Amplified PCR products were quantified by qPCR and normalized to IgG control.

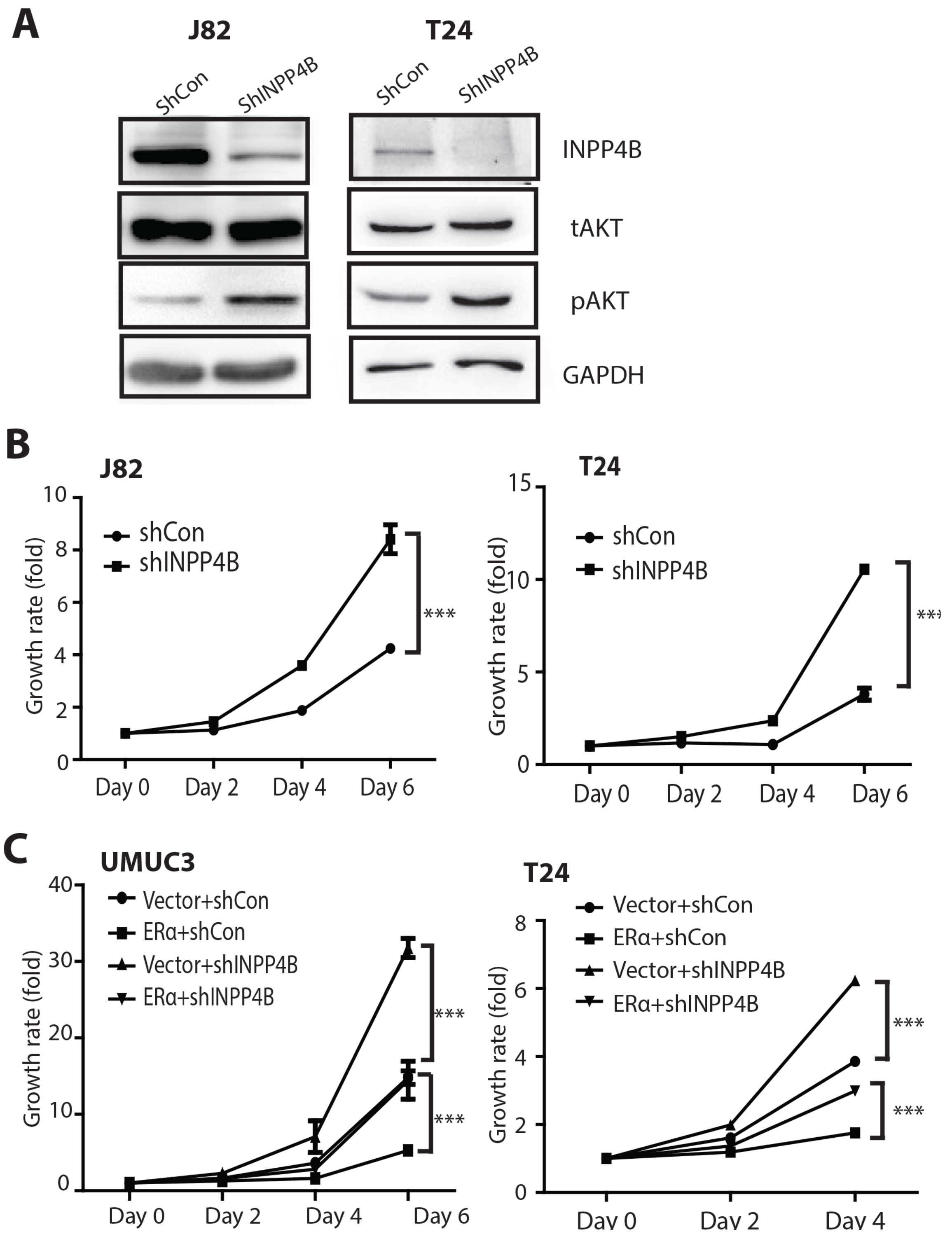

Supplemental Figure S3: Knocking down INPP4B in BCa cells results in increased AKT phosphorylation and cell growth. **(A)** Protein lysates were collected from J82 and T24 with lentiviral PLKO.1-sh-control (shCon) or PLKO.1-sh-INPP4B (shINPP4B) transductions. INPP4B, total AKT, phosphorylated AKT, and GAPDH were detected by western blotting. **(B)** J82 and T24 with lentiviral PLKO.1-sh-control (shCon) or PLKO.1-sh-INPP4B (shINPP4B) transductions were used to assay cell growth on days 0, 2, 4 and 6. **(C)** UMUC3 and T24 cells with lentiviral pWPI+PLKO.1-sh-control (vector+shCon), pWPI+PLKO.1-sh-INPP4B (vector+shINPP4B), pWPI-ERα + PLKO.1-sh-control (ERα+shCon), and pWPI-ERα + PLKO.1-sh-INPP4B (ERα+shINPP4B) transductions were subjected to MTT growth assays on days 0, 2, 4 and 6.***, $P<0.001$ by Two-way ANOVA test.

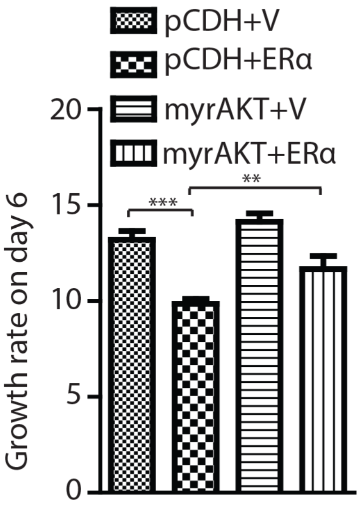

Supplemental Figure S4: Constitutively active AKT (myrAkt) expression diminished ERα growth inhibiting effect. T24 cells transfected with vector or ERα following by transfection with pCDH vector or myrAKT were analyzed by growth assays for 6 days. The transfection of ERα inhibits the BCa growth (lane 2 vs lane 1). The ectopic expressed myrAkt can reverse the ERα mediated growth inhibition (lane 4 vs. lane 2). ** $p<0.01$, *** $p<0.001$.

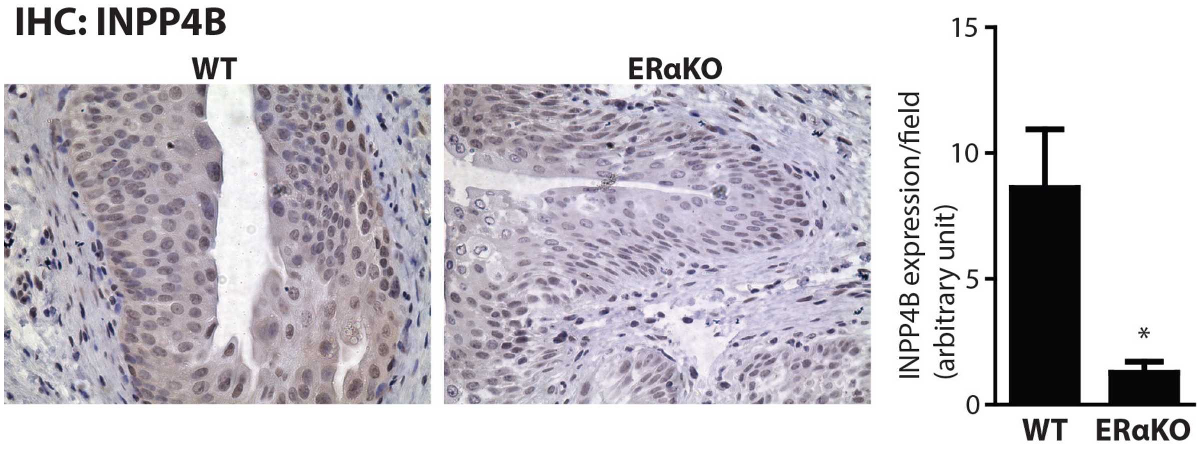

Supplemental Figure S5: Reduced INPP4B in BBN treated specific ERα knockout mouse bladder tissues. Immunohistochemical staining was performed and compared in BBN induced mouse BCa tissues from WT and UPII-ERαKO female mice. IHC was performed with antibodies against INPP4B (n=3 in WT and n=3 in ERαKO) in the bladder tissues from BBN treated WT and UPII-ERαKO female mice at 35 weeks old. p<0.05 compared to WT mice.

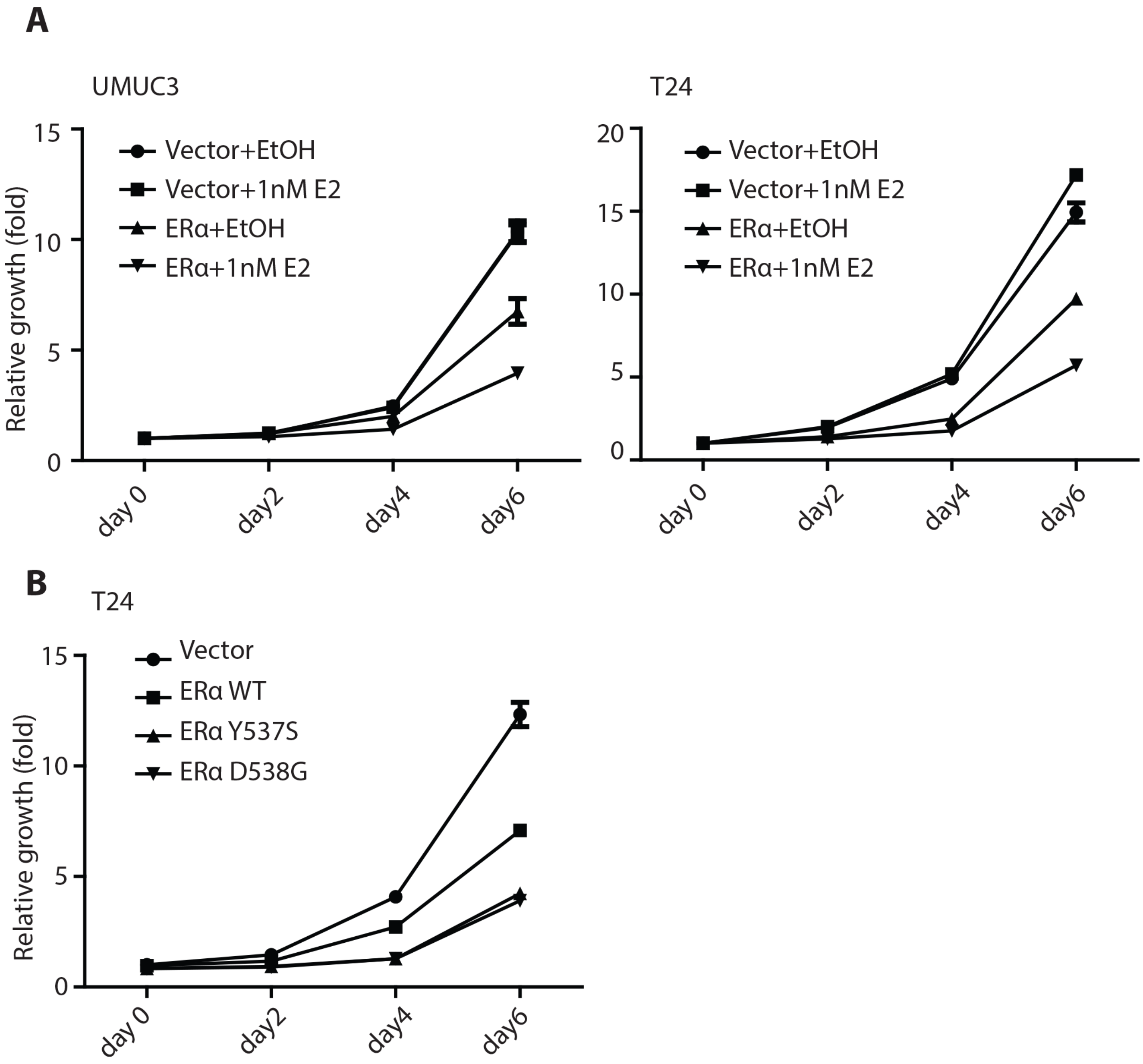

Supplemental Figure S6: Transcriptionally active ERα inhibits bladder cancer growth. **(A)** UMUC3 and T24 cells with vector or ERα expression were cultured in DMEM media supplemented with charcoal stripped FBS for 2 days and subjected to MTT growth assays under EtOH or 1 nM 17β-estradiol (E2) treatment on days 0, 2, 4 and 6. **(B)** T24 cells with vector or WT ERα or constitutive transcriptional active ERα (Y537S and D538G) expression were used to assay cell growth on days 0, 2, 4 and 6.

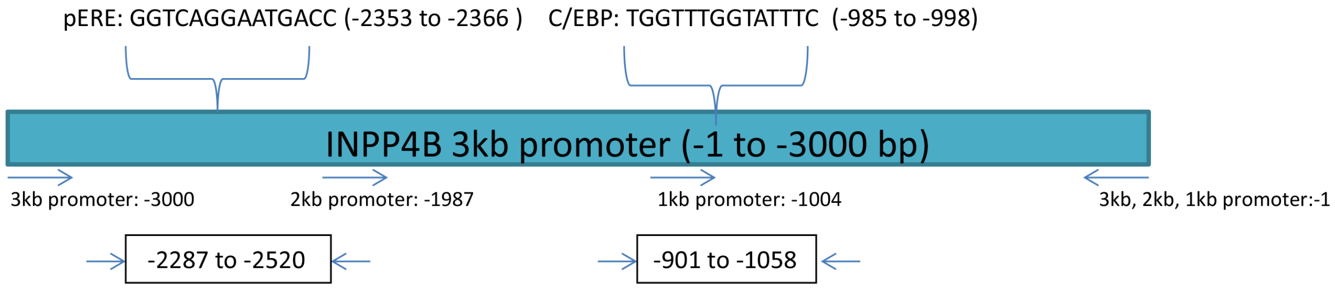

Supplemental Figure S7: Schematic representation of 3kb INPP4B promoter structure. Putative ERE binding site has been found located at -2353 to -2366 bp. C/EBP binding site is located at -985 to -998 bp. 3kb INPP4B promoter is constructed within -1 to -3kb bp. 2kb INPP4B promoter is constructed within -1 to -1987 bp. 1kb INPP4B promoter is constructed within -1 to -1004 bp. Primers designed for CHIP assay for coverage of putative ERE site are from -2287 to -2520 bp, and coverage of C/EBP binding site is from -901 to -1058 bp.

Supplemental Table S1. Primer sequences for quantitative PCR

Gene	Accession No.	Sequences
GAPDH	NM_002046	Forward: 5′-GGAGCGAGATCCCTCCAAAAT-3′<br>Reverse: 5′-GGCTGTTGTCATACTTCTCATGG-3′
C-MYC	NM_002467	Forward: 5′-TGC GTG ACC AGA TCC C-3′<br>Reverse: 5′-CGC ACA AGA GTT CCG TA-3′
ERBB2	NM_001005862	Forward: 5′-TGACACCTAGCGGAGCGAT-3′<br>Reverse: 5′-GGGGGATGTGTTTTCCCTCAA-3′
PIM1	NM_001243186	Forward: 5′-GTCCAAAATCAACTCGCTTGC-3′<br>Reverse: 5′-CCACCTGGTACTGCGACTC-3′
RB1	NM_000321	Forward: 5′-TTGGATCACAGCGATACAAACTT-3′<br>Reverse: 5′-AGCGCACGCCAATAAAGACAT-3′
C-Kit	NM_000222	Forward: 5′-CGTTCTGCTCCTACTGCTTCG-3′<br>Reverse: 5′-CCACGCGGACTATTAAGTCTGA-3′
C-MYB	NM_001130172	Forward: 5′-GAAAGCGTCACTTGGGGAAAA-3′<br>Reverse: 5′-TGTTCGATTCGGGAGATAATTGG-3′
C-MET	NM_000245	Forward: 5′-AGCAATGGGGAGTGTAAAGAGG-3′<br>Reverse: 5′-CCCAGTCTTGTACTCAGCAAC-3′
P53	NM_000546	Forward: 5′-CCGCAGTCAGATCCTAGCG-3′<br>Reverse: 5′-AATCATCCATTGCTTGGGACG-3′
INPP4B	NM_003866	Forward: 5′-GCCGAAGTTTCTTGGGCTATG-3′<br>Reverse: 5′-CTTCTATGGTGCCAACCACTTT-3′
